# Urban Particles Elevated *Streptococcus pneumoniae* Biofilms, Colonization of the Human Middle Ear Epithelial Cells, Mouse Nasopharynx and Transit to the Middle Ear and Lungs

**DOI:** 10.1038/s41598-020-62846-7

**Published:** 2020-04-06

**Authors:** Mukesh Kumar Yadav, Yoon Young Go, Indong Jun, Sung-Won Chae, Jae-Jun Song

**Affiliations:** 1 Department of Biotechnology, Pachhunga University College, Mizoram Central University, Aizawl, Mizoram 796001, India; 2 Institute for Medical Device Clinical Trials, Korea University College of Medicine, Seoul, South Korea; 3 Department of Otorhinolaryngology-Head and Neck Surgery, Korea University College of Medicine, Seoul, South Korea

**Keywords:** Microbiology, Health care

## Abstract

Air-pollutants containing toxic particulate matters (PM) deposit in the respiratory tract and increases microbial infections. However, the mechanism by which this occurs is not well understood. This study evaluated the effect of urban particles (UP) on *Streptococcus pneumoniae* (pneumococcus) *in vitro* biofilm formation, colonization of human middle ear epithelium cells (HMEECs) as well as mouse nasal cavity and its transition to the middle ear and lungs. The *in vitro* biofilms and planktonic growth of *S. pneumoniae* were evaluated in metal ion free medium in the presence of UP. Biofilms were quantified by crystal violet (CV) microplate assay, colony forming unit (cfu) counts and resazurin staining. Biofilm structures were analyzed using a scanning electron microscope (SEM) and confocal microscopy (CM). Gene expressions of biofilms were evaluated using real time RT-PCR. Effects of UP exposure on *S. pneumoniae* colonization to HMEECs were evaluated using fluorescent *in-situ* hybridization (FISH), cell viability was detected using the Ezcyto kit, apoptosis in HMEECs were evaluated using Annexin-V/PI based cytometry analysis and reactive oxygen species (ROS) production were evaluated using the Oxiselect kit. Alteration of HMEECs gene expressions on UP exposure or pneumococci colonization was evaluated using microarray. *In vivo* colonization of pneumococci in the presence of UP and transition to middle ear and lungs were evaluated using an intranasal mice colonization model. The UP exposure significantly increased (*p < 0.05) pneumococcal *in vitro* biofilms and planktonic growth. In the presence of UP, pneumococci formed organized biofilms with a matrix, while in absence of UP bacteria were unable to form biofilms. The *luxS, ply, lytA, comA, comB* and *ciaR* genes involved in bacterial pathogenesis, biofilm formation and quorum sensing were up-regulated in pneumococci biofilms grown in the presence of UP. The HMEECs viability was significantly decreased (p < 0.05) and bacteria colonization was significantly elevated (p < 0.05) in co-treatment (UP + *S. pneumoniae*) when compared to single treatment. Similarly, increased apoptosis and ROS production were detected in HMEECs treated with UP + pneumococci. The microarray analysis of HMEECs revealed that the genes involve in apoptosis and cell death, inflammation, and immune response, were up-regulated in co-treatment and were unchanged or expressed in less fold in single treatments of UP or *S. pneumoniae*. The *in vivo* study showed an increased pneumococcal colonization of the nasopharynx in the presence of UP and a higher transition of bacteria to the middle ear and lungs in the presence of UP. The UP exposure elevated *S. pneumoniae in vitro* biofilm and colonization of HMEECs, and *in vivo* mouse nasopharyngeal colonization, and increased dissemination to mouse middle ear and lungs.

## Introduction

World-wide air-pollution is a major problem due to toxic particulate matter (PM) contents that exert a negative effect on human health. In 2012 alone, approximately 7 million deaths have been reported, particularly in developing countries where pneumonia was one of the most common disease^1–4^. Urban particles (UP) are a major component of air-pollution and are a mixture of liquid and suspended atmospheric particles that consists of various size particles with an average size of 10 µm (aerodynamic diameter ≤ 10 μm, PM_10_), along with fine (PM_2.5_) and ultrafine particles (PM_0.1_)^5^. These UP also contain various metal ions such as iron, calcium, manganese, sodium, magnesium, zinc, copper etc.^6^. The UP are inhaled and deposited on various parts of body including the nose, pharynx, larynx, trachea, bronchi, and distal lungs. Epidemiological studies have revealed that vehicle emissions and cigarette smoke containing PM exposure increased the vulnerability to respiratory infections including otitis media (OM) and pneumonia^7–10^. Previously, PM including UP have been implicated in enhancing respiratory tract and nosocomial microbial infection such as OM, bronchitis, bronchiolitis, tuberculosis, atypical mycobacterial infections, pneumonia, and meningitis^11,12^.

*Streptococcus pneumoniae* is a Gram positive commensal opportunistic pathogen that colonizes the nasopharynx and is responsible for two million deaths annually^13,14^. Pneumococci initially colonizes the nasopharyngeal cavity in the form of biofilms, and from these, transits to other sterile sites causing chronic otitis media (COM), meningitis, pneumonia, bacteremia and sepsis in immune-compressed patients, children and the elderly^15,16^. The initial interaction of PM such as UP and nosocomial bacteria such as *S. pneumoniae* occurs in the nasopharyngeal cavity, and PM exposure increases the risk of infection and alters host’s defense^17,18^. Indeed, severe pneumonia and OM were reported in cigarette smoker’s exposure to PM and bacteria such as *Streptococcus* and *Haemophilus*^10,12^. The adverse effects of PM, including UP alone on human health, including pneumonia, OM and various other diseases is known^17,19–21^. Similarly various studies have reported that *S. pneumoniae* is implicated in nosocomial infections such as pneumonia, OM, meningitis and bacteria^16^. The UP and *S. pneumoniae* are the two risk factors for pneumonia and OM that interacts in the nasopharyngeal cavity. However, the interaction of *S. pneumoniae* in the presence of UP or vise-versa is not well understood. Whether the pre-exposure of UP to the nasopharynx increases pneumococcal colonization or if the presence of UP reverts commensal pneumococci to the pathogenic kind needs to be understood. We hypothesize that the presence of UP favors *S. pneumoniae* biofilm formation, colonization of epithelial cells, and the nasopharynx and risk factor AOM and pneumonia. Therefore, in this study, we evaluate the effects of UP exposure on; (i) *S. pneumoniae in vitro* biofilm and planktonic growth, (ii) pneumococcal colonization and toxicity to human middle ear epithelium cells (HMEECs) and alteration of global gene expression (iii) pneumococcal colonization of mouse nasopharynx and dissemination to the middle ear and lungs.

## Material and Methods

### Ethical statement

The animal experiments were carried out as per the Institutional Animal Care and Use Committee (IACUC) Korea University Seoul, South Korea. Animal protocols were approved by the Institutional Review Board of Korea University, college of medicine, Seoul, South Korea with IRB number KOREA -2017-0190. The HMEECs used in this study was kindly provided by Dr. David J. Lim (House Ear Institute, LA, USA).

### Bacterial strains and culture medium

*Streptococcus pneumoniae* D39 is Avery’s virulent serotype 2 (NCTC 7466) strain obtained from the Health Protection Agency Culture Collection (Salisbury, UK)^22^. It is a fully sequenced and highly virulent strain in animal models, even after many years of its isolation from a patient^23,24^. Other *S. pneumoniae* such as serotype 3 (ATCC 6303) 19A and 19F (ATCC 49619) were obtained from ATCC, USA. The antibiotic resistant strain of *S. pneumoniae* (CCARM 4001) was purchased from (culture collection of Antimicrobial resistant microorganism, Seoul, South Korea). The bacterial strains were grown on blood agar plates (BAP) supplied with 5% sheep blood purchased from a local supplier (Yang chemical, Seoul, South Korea). The UP was purchased from NIST (NIST 1604a), and stock solutions were prepared in phosphate buffered saline (PBS) and sonicated for 5 minutes and sterilized using an autoclave.

### Effect of UP on *in vitro* biofilm formation and planktonic growth

The effect of UP on *S. pneumoniae* D39 planktonic and biofilm growth was evaluated in a metal ion free medium in the presence of different concentrations of UP (50–200 µg/mL). The metal ion free medium was prepared by treating brain heart infusion (BHI) broth medium with chelex-100 (Sigma) that chelate metal ions^25^. The bacteria’s planktonic growth was detected by measuring optical density at 600 nm, resazurin staining and cfu counts at various time points. The effects of UP on *in vitro* biofilm growth of *S. pneumoniae* were evaluated using the static microtiter plate assay, resazurin staining and cfu counts^24,26^. Briefly, the bacterial colony was grown overnight on BAP that were scraped and subsequently grown in BHI medium till log-phase and pelleted by centrifugation. A 1 × 10^5^ cfu/mL cell suspension was prepared in chelex-100 treated medium, and 200 µL was inoculated in 96-well plates or 1 mL in 24-well plates. The UP solutions were vortexed and final concentrations of 50–200 µg/mL were added. The plates were incubated at 37 °C in 5% CO_2_ for 48 hr. After incubation, the medium was removed and the plates were washed twice with sterile water and stained with 0.1% CV for 15 min. The plates were washed twice and CV was dissolved in ethanol and absorbance was measured at 570 nm using a spectrophotometer. The metabolically active cells within the biofilms grown with UP (200 µg/mL) were detected by resazurin staining. Resazurin, is a non-fluorescent blue colored dye that is reduced to a pink colored fluorescence (Resorufin) by metabolically active cells allowing quantification of viable cells. Resazurin staining was performed by a procedure previously reported^27^. For resazurin staining the biofilm were washed and incubated with (0.02%) resazurin dye in 100 μL BHI-broth for 30 min at 37 °C. The fluorescence was measured (excitation 530 nm, emission 590 nm) using a multimode microplate reader (Thermo Scientific, Waltham, MA, USA). The viable bacteria within the biofilms were detected by cfu counting. The biofilms were washed and dissolved in sterile water and serially diluted and plated on BAP. The bacteria colonies were counted after over-night incubation at 37 °C.

### Scanning electron microscopic (SEM) analysis of *in vitro* biofilms

The morphology of biofilms grown in the presence of 200 µg/mL UP were evaluated using SEM. The *S. pneumoniae* D39 biofilms were grown in 24-well microplates by the procedure mentioned above for 48 hr. Biofilms were washed and fixed with 2% glutaraldehyde and 2.5% paraformaldehyde. Post-fixation, the biofilms were treated with 1% osmic acid for 2 hr, followed by dehydration by treatment with different concentration of ethanol from 60–95%. The biofilms were immediately washed three times and preserved in t-butyl alcohol at −20 °C. The biofilms were freeze dried and coated with platinum. The images were captured using a field emission scanning electron microscope (Hitachi, Tokyo, Japan).

### *In vitro* biofilms analysis via confocal microscopy (CF)

*In-vitro* biofilms grown in the presence of UP (200 µg/mL) were analyzed using confocal microscopy and fluorescence *in-situ* hybridization (FISH) by the procedure reported earlier^28^. The *S. pneumoniae* D39 biofilms were grown in μ-slides (Ibidi, Germany) for 48 hr and washed and hybridized with a peptide nucleic acid (PNA) universal bacterial probe EUB338 (5′-TGCCTCCCGTAGGA-3′)^29^. The PNA probe was labeled at the N terminus with AlexaFluor488 via a double 8-amino-3,6-dioxaoctanoic acid (AEEA) linker and was synthesized from Panagene (Dageon, South Korea). The biofilm samples were prefixed in 4% paraformaldehyde, dehydrated with ethanol and hybridized with a probe at 46 °C for 3 hr in hybridization buffer (5 M NaCl, 1 M Tris-HCl, 2% SDS and 10% formamide). The biofilms were washed in washing buffer at 48 °C. The probe labeled biofilm was examined using a Nikon A1 confocal microscope (Nikon Instruments, Inc., NY, USA) with FITC channel.

### Streptococcus pneumoniae *in vitro* biofilm gene expressions

The gene expressions of biofilm related gene (*luxS*), competence related genes (*comA, comB, ciaR*) and toxin related genes (*ply, lytA*) were evaluated using real-time RT PCR. *S. pneumoniae* D39 biofilms were grown in metal ion-free medium by procedure mentioned above, and total RNA was extracted using RNeasy Total RNA Isolation System Kit (Qiagen, Valencia, CA, USA). The RNA was quantified using Nano-drop, and cDNA was synthesized using Bioneer cDNA synthesis kit (Seoul, Korea). The cDNA amplification was carried out in a 20 µL reaction mixture consist of 10 µL 2x SYBR Green PCR mastermix, 4 µL cDNA, 2.5 pmol forward and reverse primers. The PCR reaction was performed for 45 cycles, with an initial denaturation step at 95 °C for 2 min, followed by 45 cycles of denaturation at 95 °C for 30 s and annealing and extension at 60 °C for 1 min. The primers used in real-time PCR are given in Supplementary Table 1. The 16S RNA gene was reference gene and biofilms without UP treatment was standard condition. The relative quantification of biofilm genes were carried out using 2^−ΔΔCT^ method.

### The HMEECs viability assay

The HMEECs were maintained and routinely cultured in airway epithelial cell growth medium (PromoCell GmbH, Sickingenstr Heidelberg, Germany) supplemented with bovine pituitary extract (0.004 mL/mL), epidermal growth factor (10 ng/mL), insulin (5 µg/mL), hydrocortisone (0.5 µg/mL), epinephrine (0.5 µg/mL), triiodo-L-thyronine (6.7 ng/mL), transferrin (10 µg/mL), retinoic Acid (0.1 ng/mL) and 1% fetal bovine serum and 5% CO_2_. In a 96-well plate, 1 × 10^4^ HMEECs were seeded and incubated at 37 °C with 5% CO_2_ for 24 hr. Following incubation, cells were washed with PBS and medium was replaced with UP (200 µg/mL) containing medium (antibiotic free medium) and further incubated for 8 hr. The HMEECs pre-exposed to UP were infected with *S. pneumoniae* (MOI 10) for 15 hr. The HMEECs viability was detected using the EZ-cytox cell viability kit (Dogenbio, Korea) as per manufacturer’s instruction.

### The HMEECs apoptosis detection

The apoptosis of HMEECs pre-exposed to UP followed by *S. pneumoniae* treatment were detected by the Annexin-V/PI double staining apoptosis detection kit according to the manufacturer’s protocol (BD, San Diego, CA, USA). Briefly, 1 × 10^6^ HMEECs were seeded overnight in a six-well plate and the medium was replaced with fresh medium containing UP (200 µg/mL) and incubated for 8 hr. Thereafter, the HMEECs were treated with *S. pneumoniae* (MOI 10) for 15 hr. After treatment, cells were collected and washed twice with PBS and re-suspended in 1× binding buffer [10 mM HEPES/NaOH (pH 7.4), 140 mM NaCl, and 2.5 mM CaCl_2_]. The cells were stained with Annexin-V for 15 min followed by PI staining and evaluated using a flow cytometer (Beckman Coulter; Fullerton, CA, USA). The apoptosis rate was calculated using Beckman Coulter software.

### Reactive oxygen species (ROS) detection in HMEECs

The ROS generated in HMEECs exposed to UP and *S. pneumoniae* were detected by the OxiSelect ROS assay kit (Cell Biolabs; San Diego, CA, USA) according to the manufacturer’s procedure. Briefly, 1 × 10^4^ HMEECs were grown in a 96-well dark-well clear bottom plate for 24 hr. The cells were washed twice with PBS and exposed to 100 µL of 2′,7′-dichlorofluorescein-diacetate (DCFH-DA) in culture medium at 37 °C for 40 min. The cells were treated with UP (200 µg/mL), followed by *S. pneumoniae* (MOI 10) treatment. The ROS was detected measuring fluorescence using a microplate reader at 480/530 nm. Hydrogen peroxide was used as a positive control.

### *Streptococcus pneumoniae* biofilm/aggregation on HMEECs monolayer

Pneumococcal biofilm or aggregations on HMEECs in the presence of UP were evaluated using PNA-probe fluorescent *in situ* hybridization (FISH). The PNA-probe used for bacterial detection is mentioned above in the confocal microscopy section. Briefly, 5 × 10^5^ HMEECs were seeded in a 4-chamber glass-slide with a confocal compatibility surface. The cells were grown for 24 hr and treated with UP (200 µg/mL) for 8 hr followed by *S. pneumoniae* (1 × 10^7^) treatment for 12 hr. The slides were washed and pre-fixed in 4% paraformaldehyde and hybridized with the PNA bacterial probe at 46 °C for 3 hr followed by washing. The background nuclei of HMEECs were counter stained with Hoechst dye. The cells were analyzed under a confocal microscope. The bacteria were visualized with the FITC (green) channel and the background HMEECs nuclei with the DAPI (blue) channel.

### The HMEECs global gene expression analysis using microarray

To educate the molecular mechanism underlining the elevated toxicity of UP + *S. pneumoniae*, gene expression analysis was performed using microarray. Global gene expression of HMEECs treated with UP (200 µg/mL), *S. pneumoniae* (MOI 10) or co-treatment (UP + *S. pneumoniae*) were analyzed using the Affymetrix microarray chip. The total RNA of HMEECs was extracted using the Trizol reagent (Invitrogen, USA), RNA quality was assessed using the Agilent 2100 Bioanalyzer (Agilent Technologies, USA), and quantity was determined using the ND-1000 spectrophotometer (NanoDrop Technologies, USA). The gene expression of HMEECs was detected by the Affymetrix GeneChip Human Gene 2.0 ST oligonucleotide array using the manufacturer’s procedure (http://www.affymetrix.com)^30^. The labeling and hybridization protocols used were standardized by e-biogen (e-biogen Inc., Seoul, South Korea) as per GeneChip expression analysis technical manual (Affymetrix, Inc., USA)^30^. Briefly, 300 RNA was converted to cDNA using T7-oligo (dt) promoter primer, and through *in vitro* transcription the cRNA was synthesized from cDNA using Invitrogen kit (Invitrogen, USA). The fragmented cDNA was end-labeled through terminal transfer reaction incorporating a biotinylated dideoxynucleotide and hybridized to the GeneChip Human Gene 2.0 ST array (Affymetrix, Inc., USA)^30^. The hybridization reaction was performed by incubating samples at 45 °C for 16 hr by procedures described by Affymetrix (Affymetrix Inc., USA)^30^. The hybridized and Streptavidin Phycoerythrin stain chips were scanned using Affymetrix Model 3000 G7 scanner and the raw.cel files and expression data were generated by Affymetrix Command Console software1.1 (Affymetrix Inc., USA)^30^. The expression data were normalized by Robust Multi-Average. The 2-fold change of gene expression in treated samples with respect to the control sample (untreated) was considered significant. The gene ontology (GO) and KEEG pathway analysis was performed using DAVID (http://david.abcc.ncifcrf.gov/), string and Medline databases (http://www.ncbi.nlm.nih.gov/). Microarray data were submitted to NCBI GEO with access number GSE138091. The microarray gene expressions of ten genes were differentially expressed in microarray and were confirmed by real time (RT) PCR using the procedure mentioned above (biofilm gene expression). The primers are shown in Supplementary Table I.

### *In vivo* colonization of *S. pneumoniae* in mouse nasopharynx, middle ear and lungs

*In vivo* colonization of *S. pneumoniae* in the presence of UP and subsequently transit to the middle ears and lungs was evaluated using intranasal mice models^31–33^. Twenty-seven, pathogen-free C57BL/6 mice, 10–14 weeks of age were obtained from Koatech (Pyeongtaek, South Korea). Upon arrival, mice were examined for any abnormality, and housed isolated for 2 weeks for acclimatization in an infection free zone. Animals were divide into five groups according to the treatment; vehicle control (PBS, *n* = 6), UP only (UP 400 µg/mice, *n* = 6), *S. pneumoniae* only (1 × 10^7^/mice, *n* = 6), UP + *S. pneumoniae* (*n* = 6), and untreated (without any procedure, *n* = 3). The UP was suspended in PBS, and the suspension was sonicated for 5 min before being used. Fifteen microliters of UP or bacterial suspension were intranasally administrated by dropping in mice nares without anesthesia. The mice were kept in an invert position for 5 min to pass the solution through the nasal cavity of the mice. Two hundred microgram per mice UP and 5 × 10^6^ bacteria were inoculated twice so that the final UP quantity were 400 µg/mice and bacteria were 1 × 10^7^/mice, respectively. The mice were incubated for 5 days, and were sacrificed under deep anesthesia and cervical dislodging. The nasal lavages were collected by washing the nasal cavity with PBS by inserting a cannulated needle into the trachea. Bacterial load in nasal lavages were detected by plating serially diluted nasal lavages on BAP. The lungs and both bullae were aseptically harvested. The lungs and bullae were homogenized and the lysate was serially diluted and plated on BAP. Pneumococci cfu were counted after overnight incubation at 37 °C.

### Statistical analyses

Biofilm experiments were carried out in triplicate, repeated at least three times and results from independent experiments were averaged and standard deviations were calculated. Other experiments were performed in replicates and repeated to calculate statistically significance. The mean of the replicates and the statistical significance were calculated using student’s t test and one way ANOVA and p-values less than 0.05 was considered significant.

## Results

### The presence of UP favors bacterial growth

**Figure 1:**
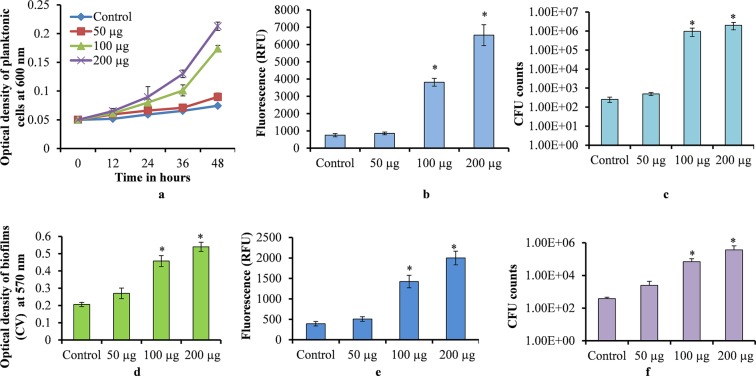
*Streptococcus pneumoniae* D39 growth in metal ion free medium in the presence of urban particles. (**a**) Time dependent plankton growth of *S. pneumoniae* detected by measuring optical density at 600 nm. (**b**) Metabolically active bacteria in 48 hr grown plankton cells detected by resazurin staining. (**c**) Cfu counts of 48 hr grown plankton bacteria. (**d**) *In vitro* biofilm (48 hr grown) biomass detected by the CV-microplate assay. (**e**) Metabolically active cells within 48 hr of biofilm detected by resazurin staining. (**f**) Viable bacteria within 48 hr of grown biofilm detected by cfu counts. The error bars represent the standard deviation from the mean value. The statistical significances were calculated using the student’s t test, and a p-value less than 0.05 was considered significant.

The time dependent planktonic growth of *S. pneumoniae* in the presence of different concentrations of UP, showed an increased cell growth in metal ion free medium supplemented with UP. Initially, the pneumococcal growth was slow, however, at 36 and 48 hr the pneumococci growth was significantly elevated (p < 0.05) in the presence of UP in comparison to the control (Fig. 1a). The metabolically active bacteria detected by resazurin staining were also increased at 48 hr. The plankton cells grown with UP (100 and 200 µg/mL) showed significantly elevated (p < 0.05) fluorescence than the control (Fig. 1b). Similarly, the cfu counts of the plankton cells after 48 hr of growth were significantly elevated (p < 0.05) in presence of UP (100 and 200 µg/ml) (Fig. 1c).

*In vitro* biofilm growth of *S. pneumoniae* in the presence of UP was elevated. Pneumococcal *in vitro* biofilm biomass was significantly increased (p < 0.05) at 100 and 200 µg/mL UP (Fig. 1d). The resazurin staining also showed significantly increased (p < 0.05) fluorescence in samples containing UP (100 and 200 µg/mL) in comparison to the control (Fig. 1e). The viable bacteria within the biofilm growth containing UP (100 and 200 µg/mL) were significantly increased (p < 0.05) in the comparison to control biofilms (Fig. 1f). The *in vitro* biofilms of different serotypes (Serotype 3, 19A, 19F and antibiotic resistant strains) were also significantly elevated (p < 0.05) in the presence of 100 µg/mL UP (Supplementary Fig. 1). These results indicate that the presence of UP increased pneumococcal plankton and *in vitro* biofilm growth.

### SEM analysis revealed compact biofilm in the presence of UP

**Figure 2:**
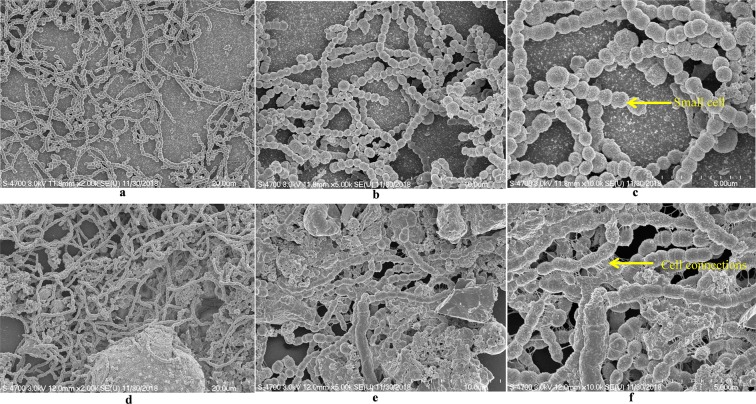
Scanning electron microscope images of *S. pneumoniae* D39 *in vitro* biofilm grown in metal ion free medium. Images (**a,b,c**) are representative images of the control biofilms (without any supplements). Images (**d,e,f**) are representative images of biofilms grown with 200 µg/mL UP. Images are 20, 10 and 5 microns.

The SEM analysis of *in vitro* biofilms grown in metal ion free medium supplemented with 200 µg/mL UP revealed a differentiated morphology in comparison to the control. In control biofilms (without any supplement), pneumococci were unable to form an organized biofilm, bacteria formed long chain like-structures, and the cell size appeared smaller than normal (pointed) with no visible matrix (Fig. 2a–c). In contrast, the biofilms grown in the presence of 200 µg/mL UP were thick and organized with presence of a matrix (Fig. 2d–f). The cells were connected to each and to the adjacent cells (pointed) and embedded in UP with presence of biofilm matrix.

### Confocal microscopy analysis of *in vitro* biofilm

**Figure 3:**
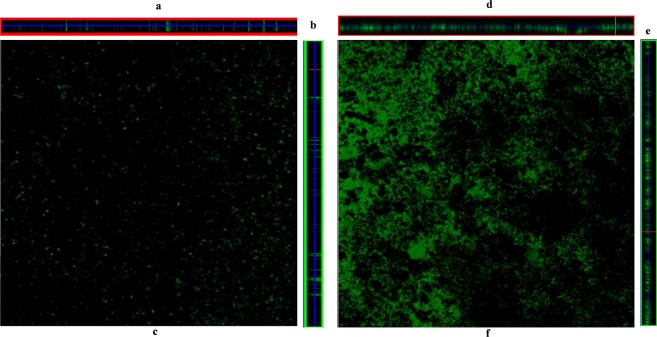
Confocal microscope images of *Streptococcus pneumoniae in vitro* biofilms grown in metal ion free medium. (**a**,**b**) are ortho, and (**c**) is a 3-dimensional confocal microscope image of biofilms grown in metal ion free medium without any supplement (control). Figures (**d**,**e**) are ortho and f is a 3-dimensional image of biofilms grown in metal ion free medium supplemented with 200 µg/mL urban particles.

The confocal microscopic analysis revealed that in the absence of UP, pneumococci were unable to formed organized biofilms. In the absence of UP, pneumococci were unable to form *in vitro* biofilms and bacteria were scattered and attached to the bottom of plate (Fig. 3c). The depth of control biofilms is shown in Fig. 3a,b (ortho images). In contrast, pneumococci formed thick biofilms with significant depth in the presence of 200 µg/mL UP (Fig. 3d,e). The cells were connected to each other and to the bottom forming a 3-D organized structure (Fig. 3f).

### Urban particles altered *Streptococcus pneumoniae* biofilm related gene expressions

**Figure 4:**
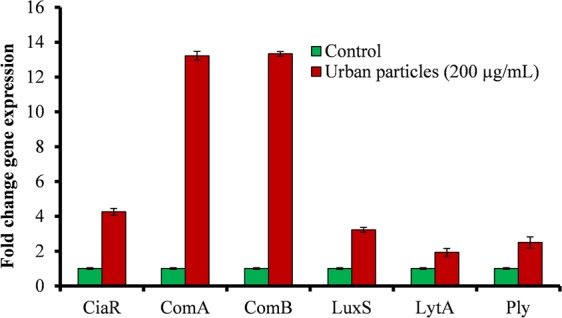
*Streptococcus pneumoniae in vitro* biofilm gene expression study using real-time RT-PCR. Graphical representation of fold change in gene expression of *S. pneumoniae* biofilms grown with 200 µg/mL urban particles with respect to the control (untreated).

The *in vitro* biofilm formation in metal ion free medium was significantly increased in the presence of UP. Therefore, to evaluate the molecular mechanism, we analyzed the gene expressions of *S. pneumoniae* biofilm grown in the presence of UP. The gene expression study showed a significant up-regulation of pneumococcal genes involved in biofilm formation, competence and toxins. Our study showed that quorum sensing (QS) and competence related genes such as *luxS*, *comA*, *comB*, and *ciaR* were up-regulated more than 2-fold in biofilms grown with 200 µg/ml UP (Fig. 4). Similarly, the *lytA* and *ply* genes involved in pathogenesis and toxin release were also up-regulated in presence of the UP (Fig. 4).

### The HMEECs exposed to UP and pneumococcal treatment decreased cell viability and increased apoptosis and ROS production

**Figure 5:**
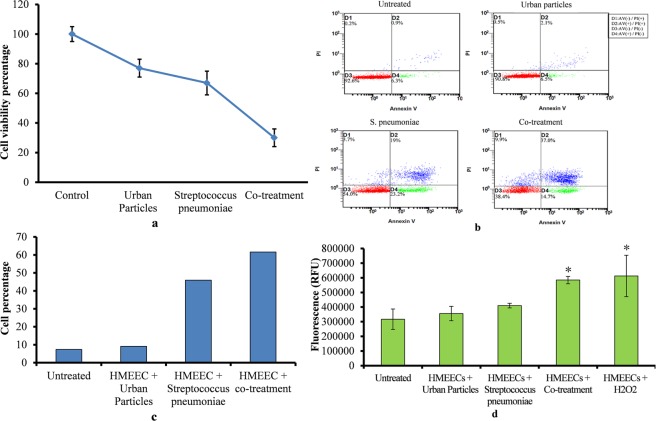
HMEECs viability, apoptosis and reactive oxygen species production in presence of UP (200 µg/ml) or *S. pneumoniae* (MOI 10) or Co-treatment. (**a**) HMEECs viability upon UP or *S. pneumoniae* or Co-treatment. (**b**) Apoptosis detection using flow cytometry analysis of Annexin V/PI stained HMEECs treated with UP only or *Streptococcus pneumoniae* only or co-treatment (UP + *Streptococcus pneumoniae*. (c) Graphical representation of HMEECs percentage showed apoptosis. (**d**) ROS production detection in HMEEC treated with UP or *Streptococcus pneumoniae* or co-treatment. The error bars representing standard deviation from mean. The statistically significant were calculated by one-way ANOVA test and p-value less than 0.05 were considered significant.

The HMEECs treatment with UP and *S. pneumoniae* showed significant decreased cell viability (Fig. 5a). The HMEECs viability in UP only or *S. pneumoniae* single treatment were approximately 75% and 65% respectively. Whereas, the viability of HMEECs in co-treatment decreased to approximately 20%.

The flow cytometry analysis of HMEECs stained with Annexin V–FITC/PI double staining showed that UP or *S. pneumoniae* single treatment caused apoptosis. However, the rate of apoptosis was significantly higher in co-treatment (Fig. 5b). In UP treatment 8.6% cells showed early and 2.6% cells showed late apoptosis. While HMEECs treated with *S. pneumoniae* showed 42.2% early & 22.7% late apoptosis (Fig. 5b). However, the early and late apoptosis in co-treatment were 51.7 and 46.9% respectively. The early and late apoptosis in HMEECs upon UP or *S. pneumoniae* single treatment were 9.1% and 45.9% respectively. However, in co-treatment the apoptotic cells increased to 61.6% (Fig. 5c).

The ROS detection assay showed increased ROS production by HMEECs on UP or *S. pneumoniae* exposure. However, the ROS produced in co-treatment was significantly higher (p < 0.05) when compared to single treatment of UP or *S. pneumoniae* (Fig. 5d). Overall, these results indicate that HMEECs death in co-treatment could be due to increased apoptosis and ROS production induction by UP and *S. pneumoniae* when inoculated together.

### *Streptococcus pneumoniae* and UP co-treatment increased bacteria colonization of HMEECs

**Figure 6:**
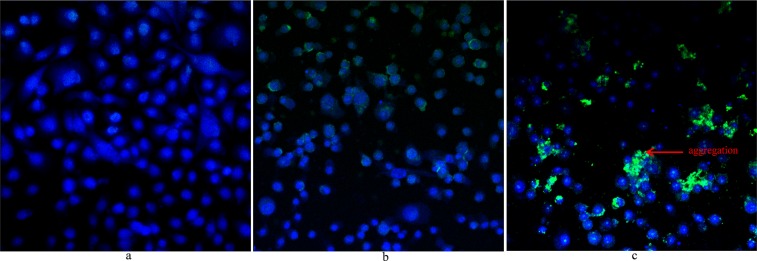
*Streptococcus pneumoniae* D39 aggregation on HMEECs monolayer detected by PNA probe and fluorescence *in situ* hybridization (FISH). (**a**) Confocal microscopic images of HMEECs untreated, cell nuclei stained with Hoechst dye were visible blue. (**b**) Confocal microscopic image of HMEECs treated with *S. pneumoniae* only, bacteria were labelled with PNA probe (green) on HMEECs and on the bottom surface. (**c**) Confocal microscopic image of HMEECs treated with UP + *S. pneumoniae* (co-treatment), bacteria formed aggregations (green) on HMEECs and were labeled with PNA probe. Images are 20×.

The effects of UP on *S. pneumoniae* aggregation/biofilm formation on HMEECs mono-layer was analyzed using FISH and confocal microscopy. The FISH results are shown in Fig. 6. In untreated HMEECs, the nuclei stained with Hoechst dye were blue (Fig. 6a). In *S. pneumoniae*-only treatment group, bacterial cells (florescent green) were attached to the bottom of the surface and on the HMEECs. However, large cell aggregates or clumps were absent (Fig. 6b). The HMEECs pre-exposed to UP and treated with *S. pneumoniae* showed large bacterial aggregates or clumps (green) (Fig. 6c). In Fig. 6, HMEECs monolayer were disrupted, probably due to cytotoxicity of UP, *S. pneumoniae* or both and cells were detached and washed during the hybridization and washing process.

### Global gene expression of HMEECs

**Figure 7:**
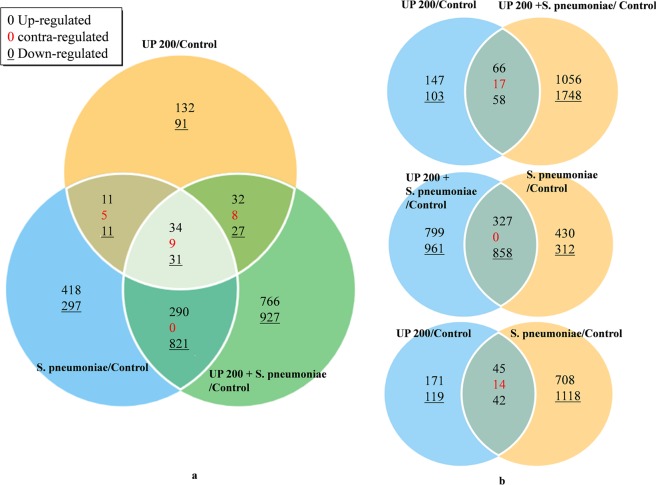
Venn diagrams illustrating differential gene expression in HMEECs treated with urban particle, *S. pneumoniae* or co-treatment with respect to the control (untreated). The global gene expression analysis was performed using total RNA microarray analysis of HMEECs. (**a**) Differentially expressed gene in all three treatments with respect to untreated cells. (**b**) Venn diagram showing differentially expressed genes in HMEECs treated with UP, *S. pneumoniae* or co-treatment.

Microarray analysis revealed 391 genes (209 upregulated and 160 down-regulated) that were significantly differentially regulated in HMEECs treated with UP (Fig. 7a). The HMEECs treated with *S. pneumoniae* showed 1927 genes (753 upregulated and 1160 down-regulated) that were significantly differentially regulated. However, HMEECs treated with UP + *S. pneumoniae* revealed 2945 genes (1122 upregulated and 1806 down-regulated) that were significantly differentially regulated (Fig. 7a). Interestingly, our microarray gene expression study revealed that only 124 genes were commonly expressed (co-expressed) in HMEECs co-treatment and UP, while a large number of genes were either expressed only in co-treatment (2804) or urban particles (250) (Fig. 7b). The gene expression study using microarray revealed that the total number of genes affected by co-treatment were higher than a single treatment of UP or *Streptococcus pneumoniae*. The gene expression analysis of ten genes differentially expressed in microarray were tested with real time PCR and the results were in agreements with the microarray results (Table III).

The KEGG pathway analyses of differentially regulated genes are shown in Supplementary Fig. 2. The KEGG pathway analysis revealed that genes involved in prostate cancer, adherens junction, Notch signaling pathway, pathways in cancer, glycerolipid and glycerophospholipid metabolism were exclusively differentially regulated in co-treatment. Whereas genes involved in legionellosis, salmonella infection, estrogen signaling pathway, spliceosome, transcriptional mis-regulation in cancer, antigen processing and presentation, and toxoplasmosis were exclusively differentially expressed in *S. pneumoniae* treatment. Whereas genes involved in cytokine-cytokine receptor interactions, arachidonic acid metabolism, and viral carcinogenesis were exclusively expressed in UP treatment. The JAK-STAT signaling pathway genes were expressed in UP and co-treatment. The MAPK signaling pathway, microRNAs in cancer and FoxO signaling genes were expressed in *S. pneumoniae* and co-treatments. A large number of genes differentially expressed in HMEECs treated with UP + *S. pneumoniae* indicates that co-treatment influences number of cellular processes and pathways.

The gene ontology analysis of differentially expressed genes in all three treatments are shown in Supplementary Fig. 3. The GO analysis showed that some gene categories exclusively expressed in co-treatment were not expressed in UP or *S. pneumoniae*. For example, the genes involve in angiogenesis and DNA repair were exclusively expressed in co-treatment and *S. pneumoniae* treatment. Similarly, the RNA splicing related genes were differentially expressed in co-treatment and not expressed in UP and only up regulated in *S. pneumoniae* treatment. Genes involved in apoptosis, cell cycle, immune response and inflammatory response, cell repair, cell growth, cell differentiation, cell proliferation, etc. were differentially expressed in all three treatments (Supplementary Fig. 3a–c). The number of genes involved in apoptosis and cell death that were upregulated in co-treatment, UP and *S. pneumoniae* were 32, 10 and 22 respectively, and the number of genes that were down-regulated were 61, 9 and 14 respectively (Supplementary Fig. 3a–c). The number of genes involved in immune responses that were up-regulated in co-treatment, UP and *S. pneumoniae* were 26, 8 and 28 respectively, while number of genes down-regulated were 57, 10, and 15 respectively (Supplementary Fig. 3a–c). The number of genes related to inflammatory response that were up-regulated in co-treatment, UP and *S. pneumoniae* were 4, 1 and 8 respectively, while the number of genes down-regulated in co-treatment, UP and *S. pneumoniae* were 14, 1 and 3 respectively (Supplementary Fig. 3a–c). Similarly, the number of genes related to cell cycle that were up-regulated in co-treatment, UP and *S. pneumoniae* were 14, 3, and 9 respectively, while the number of genes up-regulated were 66, 2, and 24 respectively (Supplementary Fig. 3a–c). Interestingly, genes involved in DNA repair were differentially regulated in co-treatment, UP and *S. pneumoniae* were 23, 1 and 12 respectively. The important genes involved in apoptosis and cell death, immune response and inflammatory responses that were significantly expressed in co-treatment and respectively expressed in single treatment of UP or *S. pneumoniae* are shown in Supplementary Table II. In co-treatment, the important genes involved in apoptosis and cell death, inflammation and immune responses that were upregulated includes Il 24, HMOX1, PTGS2, AIFM2, CASP4 (Supplementary Table II). While genes down regulated were BCL2L11, NOTCH1, IFI6, CD44, CXCL10, CIT and MKI67 (Supplementary Table II). A large number of genes differentially expressed in HMEECs treated with UP + *S. pneumoniae* indicates that inflammatory and toxicity of co-treated was significantly higher when compared to single treatment of UP or *S. pneumoniae*.

### Urban particles increased *Streptococcus pneumoniae* colonization and increased transition to the lungs and middle ear

**Figure 8:**
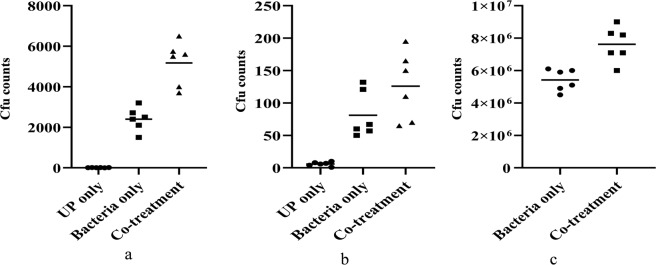
*In vivo* colonization of *Streptococcus pneumoniae* in the presence of urban particles and transition in mice middle ear and lungs (**a**) Cfu counts of *Streptococcus pneumoniae* in mice nasal lavages inoculated with *Streptococcus pneumoniae* only or UP + *S. pneumoniae*. (**b**) Cfu counts of mice middle ear inoculated with *Streptococcus pneumoniae* only or *S. pneumoniae* + UP. (**c**) Cfu counts of pneumococci in mice lungs inoculated with *S. pneumoniae* only or UP + *S. pneumoniae*. Statistical significance were calculated by students t test and a p value <0.05 was considered statistically significant.

*In vivo* mice experiment results showed an elevated colonization of pneumococci in the presence of UP in the nasopharynx of mice and a significant number of bacteria disseminated to the lungs and middle ear. The cfu counts of nasal lavages were significantly increased (p < 0.05) in mice exposed to UP and bacteria (Fig. 8a). The mean cfu counts of nasal lavages of mice inoculated with bacteria only were 2.40 × 10^3^ ± 854. Co-treatment inoculated mice nasal lavages were 5.18 × 10^3^ cfu ± 1738. Fifty-three percent more bacteria were recovered from nasal lavages in co-treatment when compared to the bacteria only treated group.

The bacterial counts in mice middle ear (bullae) revealed that a marked number of bacteria transited to the mice middle ear. The cfu counts of mice bullae inoculated with bacteria only were 1.02 × 10^2^. However, the cfu counts of bullae inoculated with UP + *S. pneumoniae* were 1.60 × 10^2^. The cfu counts of pneumococci in mice bullae inoculated with UP + *S. pneumoniae* were elevated by 30% (Fig. 8b).

Similarly, the pneumococcal counts in mice lungs revealed a significantly high (p < 0.05) number of bacteria that were able transit to the lungs in the presence of UP. In the presence of UP 22% more pneumococci were detected in mice lungs inoculated with UP and bacteria (Fig. 8c). These results indicate that the presence of UP increased the pneumococcal colonization in the nasopharynx and bacterial transit to the middle ear and lungs also increased.

## Discussion

*S. pneumoniae* asymptotically colonizes the nasal cavity and causes infections such as OM and pneumonia in immune-compromised individuals. Similarly, UP are inhaled during respiration and deposited in the nasal cavity. It was previously detected that cigarette smokers exposed to PM are more susceptible to microbial infection, including OM, which has been associated with microbial biofilms^12,20^. However, the biofilm formation and colonization of *S. pneumoniae* in the presence of UP is not well understood. In this study, we evaluated the effects of UP on *S. pneumoniae in vitro* biofilm formation, colonization of HMEECs and the nasopharynx and transit to the middle ear and lungs.

Here, we detected an elevated pneumococcal planktonic growth in the presence of UP, indicating that the UP composition induced a biological effect that favored pneumococcal growth. Planktonic growth was examined in metal ion free medium, which are critical components of bacterial growth medium. These UP contain various metals such as iron (3.92%), sodium (4240 mg/kg), silicon (12.8%), zinc (4800 mg/kg), magnesium (0.81%), manganese (790 mg/kg), calcium (5.84%), etc. (NIST, USA). The increased growth in samples supplied with UP potential indicate that pneumococcal growth was attributed to the metal components of UP. Iron is an essential nutrient for bacterial growth and many studies reported it crucial for pneumococcal energy production, nucleotide synthesis, and regulation of gene expression^34^. Furthermore, the *in vitro* biofilm growth results revealed elevated pneumococcal biofilm growth in the presence of UP, and bacteria formed thick biofilms that were organized in a 3–dimensional honey-comb structure as previously reported^35^. Interestingly, in the absence of UP (control) the bacteria were unable to form organized biofilms and the bacterial cell appeared small in size and formed a chain like structure. These results indicate that probably in the absence of UP, normal bacterial growth was disrupted, and in the presence of UP, pneumococci were able to maintain normal grown^34^. The primary reason for elevated biofilm growth is due to an increase in planktonic cell growth, and the second reason could be that UP provides favorable scaffold for biofilm growth^31^. In addition, the elevated expression of biofilm related genes such as *luxS*, *comA*, *comB*, *ciaR* in the presence of UP was attributed to increased biofilms. Pneumococcal biofilm formation is regulated by LuxS mediated quorum sensing (QS) and two component QS^24,34,36^. Here, our results demonstrated increased expression of *LuxS*, *ciaR*, *comA*, *comB* in biofilms grown in the presence of UP, which contain a number of metals including iron (3.92%). These results indicate that the metal contents of UP induced expressions of biofilm and competence related gene expression and elevated biofilms growth. Previously, various studies reported that the presence of metal ions, such as iron and zinc, elevated pneumococcal biofilm growth, and iron up-regulated *luxS* gene expressions and controlled biofilm formation and fratricide^25,34,37^. Overall, these results indicate that *S. pneumoniae* forms elevated biofilm growth in the presence of UP and regulated gene expression of genes involved in biofilm, competence and toxin production.

The results of this study showed decreased HMEECs viability, increased apoptosis, ROS production and increased bacteria colonization/aggregation to HMEECs in the presence of UP. *S. pneumoniae* produces various toxins including pneumolysin (encoded by the ply gene) that induces DNA damage and cell cycle arrest, and hydrogen peroxide mediated by pneumococcal autolysin LytA^38,39^. We detected up-regulation of *ply* and *lytA* genes in pneumococcal biofilms in the presence of UP. Similarly, the PM contents of UP also exerts cytotoxicity through oxidative stress, DNA oxidative damage, mutagenicity, and stimulation of pro-inflammatory factors^19,40,41^. These results indicate that UP and *S. pneumoniae* treatment individually are toxic to epithelium cells, which is mediated by elevated ROS production, and in co-treatment, ROS production was amplified^42,43^. Possibly, various factors including elevated ROS, hydrogen peroxide and toxins produced by pneumococci, increased the apoptosis rate in HMEECs^43^. Overall, these results indicate that exposure of HMEECs to UP increased cell susceptibility to *S. pneumoniae* infection, and amplified inflammation, ROS production and apoptosis that results in decreased HMEECs viability.

Global gene expression using microarray revealed up-regulation of a number of genes involved in apoptosis, inflammation and cell death. Down-regulation of genes involved in DNA repair, anti-apoptosis and cell growth in co-treatment indicates that UP + *S. pneumonia* treatment was more toxic to HMEECs. In our previous study, we detected a concentration dependent decreased HMEECs viability and increased inflammatory related gene expression in the presence of UP^19,21,41^. Here, we detected the up-regulation of pro-inflammatory cytokines such as Il 24, HMOX1 and PTGS2 in co-treatment indicated an increased inflammation^44–46^. The down-regulation of CD44 in co-treatment indicated that immune responses and defense was suppressed. It has been reported that a defect in CD44 gene expression obstruct bacteria clear during otitis media^47^. The up-regulation of apoptosis related genes (AIFM2, CASP4) and down-regulation of anti-apoptosis genes (BCL2L11, NOTCH1 and IFI6) in HMEECs indicates that co-treatment induced apoptosis in HMEECs. The AIFM2 gene encodes an apoptosis inducing factor mitochondrion associated protein, which plays a vital role in the caspase-independent cell death pathway^48^. In addition, the CASP4 protein plays an important role in the clearance of bacterial toxins, and the down-regulation may hamper bacterial clearance^49^. The BCL2L11 gene encodes for anti-apoptosis proteins that play a vital role in apoptosis and is associated with OM^50^. Similarly, the NOTCH1 gene down-regulation has been reported to induce apoptosis and inhibit cell proliferation and metastasis in laryngeal squamous cells and is a risk factor for AOM^51^. The IFI6 gene encodes protein that negatively regulates apoptosis and has been down-regulated on virus infection^52^. Another important gene down-regulated in co-treatment was CXCL10, which encodes an important anti-microbial peptide (AMP) capable of directly killing bacteria and can act as a chemoattractant for natural killer cells^53,54^. The down-regulation of CXCL10 indicates decreased defense of HMEECs. Here, our results showed up-regulation of five genes that encode metallothionein proteins in co-treatment and less than 2 fold expressed in UP or *S. pneumoniae* treatment. The metallothioneins are stress response proteins induced during inflammation, bacterial infection, restraint stress and agents that generate reactive oxygen species^55^. In addition, the cell growth related genes were also reduced as suggested by down-regulation of CIT and MKI67. The significantly low viability of HMEECs in co-treatment could be due to the amplified inflammation, which may result in HMEECs injury and resulting in cells becoming more susceptible to pneumococci infection and cell death (Supplementary Fig. 4).

A significant percentage of the population carries pneumococci asymptomatically in the nasopharynx, and after inhalation the UP find their path in the nasopharynx where they interact with epithelium cells and bacteria. Previous studies reported enhance respiratory tract and nosocomial microbial infection including OM and pneumonia in the presence of PM containing air-pollution^11,12,56^. Our *in vivo* results showed increased pneumococcal colonization in the mouse nasopharynx in the presence of UP. Probably the presence of UP decreased the host defense and obstructed bacterial clearance that favored pneumococcal colonization^57^. *S. pneumoniae* is known to form biofilms *in vivo* during nasopharyngeal and middle ear colonization^58,59^. Moreover, elevated recovery of pneumococci in the presence of UP from the nasal lavage indicated that perhaps the UP provided a favorable surface for pneumococci colonization/biofilms^31^. Biofilms in the nasopharynx act as a reservoir of bacteria, and planktonic bacteria from those biofilms can transit to other typically sterile anatomical sites such as middle ear causing OM, in blood causing bacteremia, or meningitis, and in lungs causing pneumonia^60–63^. Here, our results demonstrate that UP impacts *in vivo* colonization of pneumococci and promotes the spread from nasopharynx to the middle ear and the lungs in mouse intranasal model. Recovery of increased pneumococci in middle ear and lungs suggest that UP induces *S. pneumoniae* dissemination and subsequently colonization to the middle ear or lungs, and that could be a key factor in how PM induced microbial infections such as pneumonia and OM^3,64^.

## Conclusion

The results of this study showed that UP increased pneumococci *in vitro* biofilms and planktonic growth. Pre-exposure of HMEECs to UP decreased cell viability, increased apoptosis, ROS production and bacteria aggregation on HMEECs. The co-treatment impacts a number of genes involved in inflammatory response, apoptosis, immune responses and cell death. The presence of UP increased pneumococcal *in vivo* colonization of the nasopharynx and dissemination to the middle ear and lungs.

## Supplementary information

Supplementary figure 1 .

Supplementary figure 2.

Supplementary figure 3.

Supplementary figure 4.

Supplementary table I.

Supplementary table II.

Supplementary table III.
