# Supplementary figures and images for: Urban Particles Elevated Streptococcus pneumoniae Biofilms, Colonization of the Human Middle Ear Epithelial Cells, Mouse Nasopharynx and Transit to the Middle Ear and Lungs
Source: Sci Rep. 2020 Apr 6;10:5969. doi: 10.1038/s41598-020-62846-7 (PMC7136263; doi:10.1038/s41598-020-62846-7)

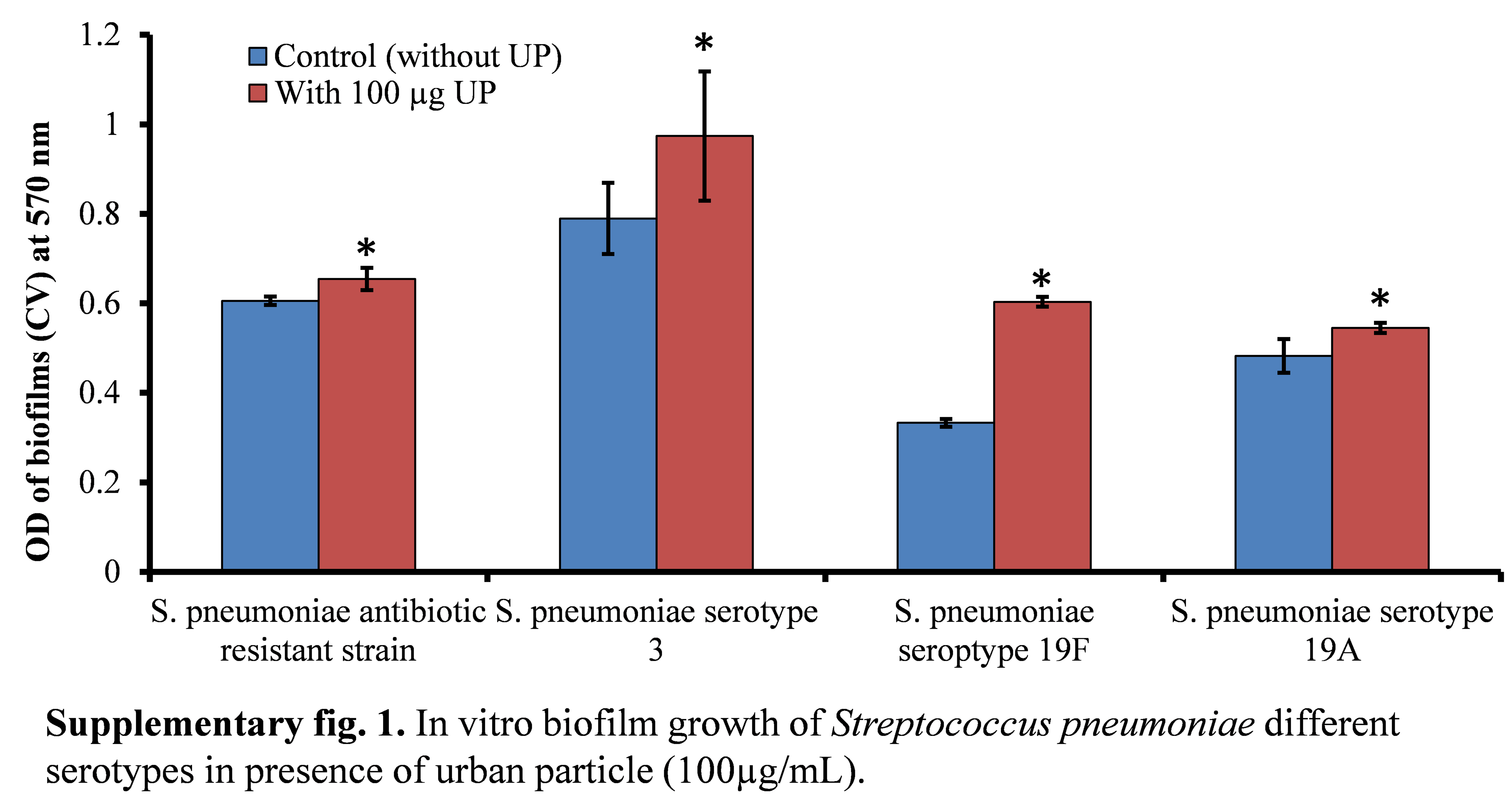

Supplement: Supplementary file 1 — Supplementary figure 1 . [file 41598_2020_62846_MOESM1_ESM.tif]

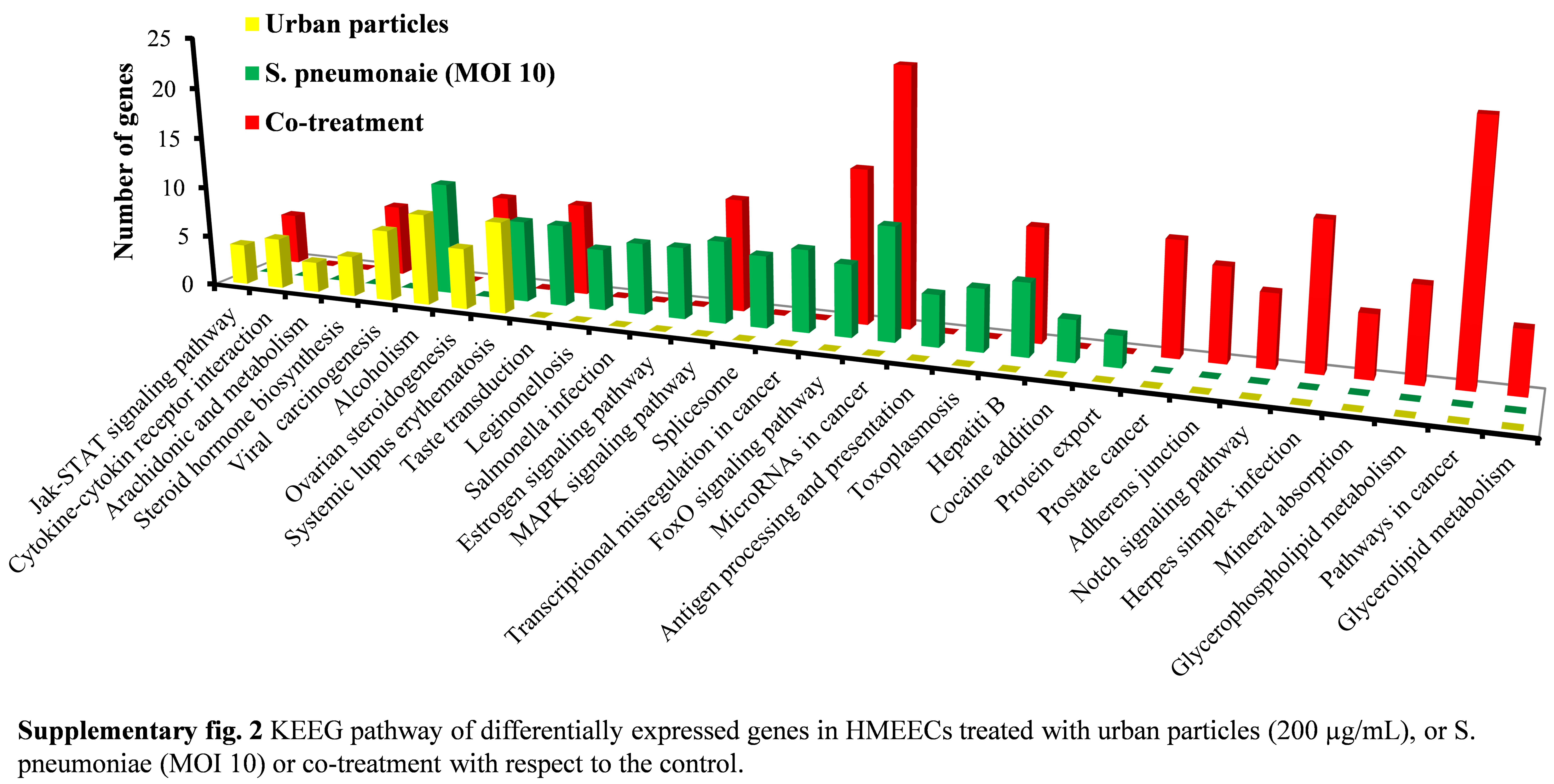

Supplement: Supplementary file 2 — Supplementary figure 2. [file 41598_2020_62846_MOESM2_ESM.tif]

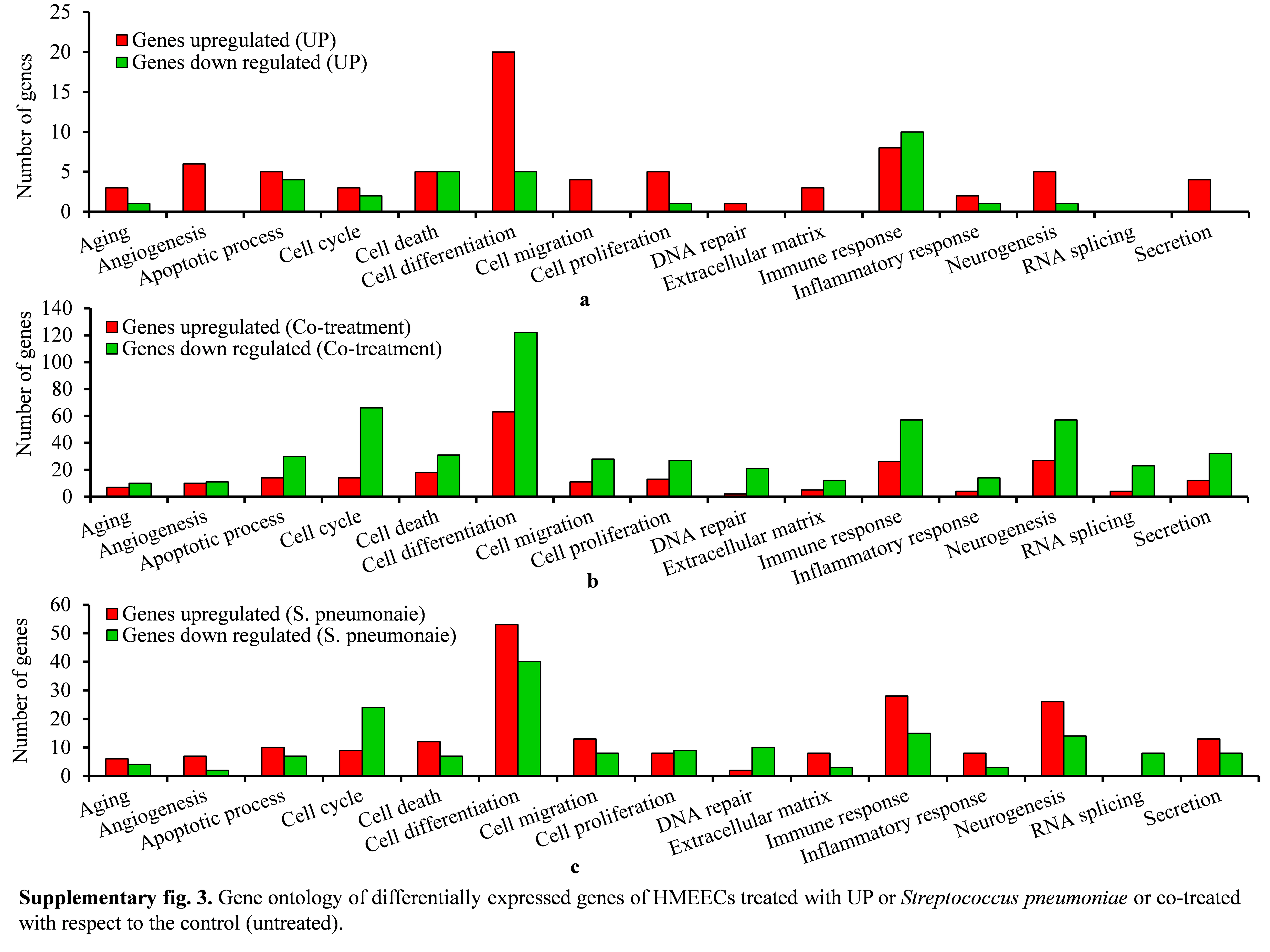

Supplement: Supplementary file 3 — Supplementary figure 3. [file 41598_2020_62846_MOESM3_ESM.tif]

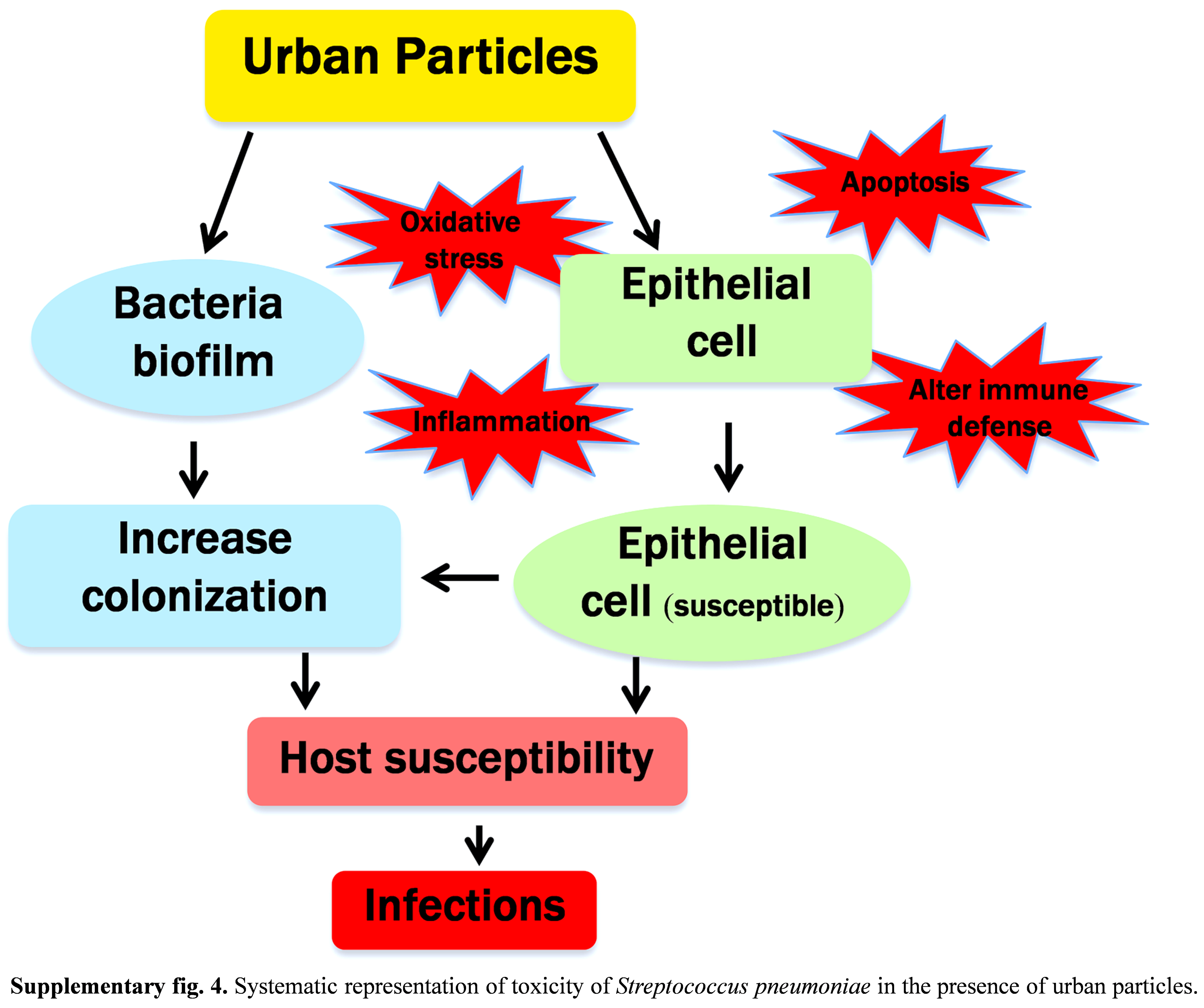

Supplement: Supplementary file 4 — Supplementary figure 4. [file 41598_2020_62846_MOESM4_ESM.tif]
