# Supplementary material for: Urban Particles Elevated Streptococcus pneumoniae Biofilms, Colonization of the Human Middle Ear Epithelial Cells, Mouse Nasopharynx and Transit to the Middle Ear and Lungs
Source: Sci Rep. 2020 Apr 6;10:5969. doi: 10.1038/s41598-020-62846-7 (PMC7136263; doi:10.1038/s41598-020-62846-7)
Supplement: Supplementary file 5 — Supplementary table I. [file 41598_2020_62846_MOESM5_ESM.docx]

SUPPLEMENTRY TABLE I. List of Genes Used for RT PCR Gene Expression.

| **Gene name** | **Primer sequence** | **Base pair** | **Amplicon** |
| --- | --- | --- | --- |
| *16s* | 5ʹ-AACCAAGTAACTTTGAAAGAAGAC-ʹ3 | 25 | 126 |
|  | 5ʹ-AAATTTAGAATCGTGGAATTTTT-ʹ3 | 25 |  |
| *luxS* | 5ʹ- ACATCATCTCCAATTATGATATTC-3ʹ | 24 | 257 |
|  | 5ʹ- GACATCTTCCCAAGTAGTAGTTTC-3ʹ | 24 |  |
| *comA* | 5ʹ- GAGACGCGAGCCATTAAGG-3ʹ | 20 | 156 |
|  | 5ʹ- GGGATCTGGATCGGCAATATGA-3ʹ | 21 |  |
| *comB* | 5'-GAACCCAGTCGTATCCTTGC-3' | 20 | 95 |
|  | 5'-TCCCCCTTCTTAACCAGCTT-3' | 20 |  |
| *ciaR* | 5'-GATGTTATGCAGGTATTTGATG-3ʹ | 20 | 157 |
|  | 5'-TAATCAGAACTGGTGTCGTAAT-3ʹ | 23 |  |
| *ply* | 5'-TGAGACTAAGGTTACAGCTTACAG-3ʹ | 23 | 225 |
|  | 5'-CTAATTTTGACAGAGAGATTACGA-3ʹ | 24 |  |
| *lytA* | 5'-CGTCCCAGGCACCATTATCA-3ʹ | 20 | 95 |
|  | 5'-CTGGCGGAAAGACCCAGAAT-3ʹ | 20 |  |
| *GAPDH* | 5´-TCGCCCCACTTGATTTTGG-3´ | 20 | 105 |
|  | 5´-GCAAATTCCATGGCACCGT-3´ | 20 |  |
| *SOD1* | 5´- GGAGACTTGGGCAATGTGAC-3´ | 20 | 147 |
|  | 5´- TCCACCTTTGCCCAAGTCAT-3´ | 20 |  |
| HMOX1 | 5′- ATTCTCTTGGCTGGCTTCCT-3′ | 20 | 124 |
|  | 5′- TGTGCTTTTCGTTGGGGAAG-3′ | 20 |  |
| PTGS2 | 5′- AGGAGGTCTTTGGTCTGGTG-3′ | 20 | 117 |
|  | 5′- ACTGCTCATCACCCCATTCA-3′ | 20 |  |
| IL24 | 5′- ATCGTGTCACAACTGCAACC-3′ | 20 | 117 |
|  | 5′- AGCTGCTTCTACGTCCAACT-3′ | 20 |  |
| MKI67 | 5′- ACCTGACAGACCTCAAGAGC-3′ | 20 | 101 |
|  | 5′- CTTTGGTGCCTTGGCATGAT-3′ | 20 |  |
| CXCL10 | 5′- GTGGCATTCAAGGAGTACCTC-3′ | 19 | 189 |
|  | 5′- TGATGGCCTTCGATTCTGGATT-3′ | 19 |  |
| CSF1 | 5′- AGGAGTGAAAGAACCCTGCA -3′ | 20 | 137 |
|  | 5′- CTTTACGGGAAATCAGGCCG -3′ | 20 |  |
| CYP1B1 | 5′- GCCTTCTTTTCCGCAGAGAG-3′ | 18 | 115 |
|  | 5′- AACCGCAACTTCAGCAACTT-3′ | 18 |  |
| IFI6 | 5′- CTGGTCTGCGATCCTGAATG-3′ | 20 | 184 |
|  | 5′- AGAGGTTCTGGGAGCTGCTG-3′ | 20 |  |
| BCL2L11 | 5′- CATTGCAGTTGTTCAGGGCT-3′ | 21 | 150 |
|  | 5′- AAGCACAGGAAGTTGCACAG-3′ | 22 |  |
