# Supplementary material for: Urban Particles Elevated Streptococcus pneumoniae Biofilms, Colonization of the Human Middle Ear Epithelial Cells, Mouse Nasopharynx and Transit to the Middle Ear and Lungs
Source: Sci Rep. 2020 Apr 6;10:5969. doi: 10.1038/s41598-020-62846-7 (PMC7136263; doi:10.1038/s41598-020-62846-7)
Supplement: Supplementary file 6 — Supplementary table II. [file 41598_2020_62846_MOESM6_ESM.docx]

SUPPLEMENTRY TABLE II. Apoptosis and Cell Death, Inflammation, Immune Responses Related Genes Significantly Differentially Regulated in HMEECs Treated with *Streptococcus pneumoniae* + UP or Non-significantly in UP or *S. pneumoniae* Only.

| **Gene name** | **Protein function** | **Fold change UP** | **Fold change S. pneumoniae** | **Fold change Co-treatment** |
| --- | --- | --- | --- | --- |
| **Apoptosis and cell death** | | | | |
| UBE4B | ubiquitination factor E4B | 1.141 | 0.720 | 0.498 |
| IL24 | interleukin 24 | 2.570 | 1.180 | 3.968 |
| TNFRSF9 | tumor necrosis factor receptor superfamily, member 9 | 0.476 | 0.596 | 0.350 |
| IFI6 | interferon, alpha-inducible protein 6 | 0.470 | 0.662 | 0.405 |
| GCLM | glutamate-cysteine ligase, modifier subunit | 1.370 | 1.762 | 2.255 |
| NOTCH2 | notch 2 | 0.942 | 0.495 | 0.471 |
| PTEN | phosphatase and tensin homolog | 1.016 | 0.231 | 0.444 |
| AIFM2 | apoptosis-inducing factor, mitochondrion-associated, 2 | 1.156 | 1.621 | 2.038 |
| FGFR2 | fibroblast growth factor receptor 2 | 0.692 | 0.506 | 0.398 |
| FGF4 | fibroblast growth factor 4 | 1.689 | 1.930 | 2.866 |
| CASP4 | caspase 4 | 1.408 | 1.496 | 2.053 |
| BLID | BH3-like motif containing, cell death inducer | 2.991 | 0.642 | 0.418 |
| ATN1 | atrophin 1 | 1.102 | 1.053 | 0.436 |
| ESPL1 | extra spindle pole bodies like 1, separase | 0.787 | 0.521 | 0.412 |
| APAF1 | apoptotic peptidase activating factor 1 | 0.714 | 0.599 | 0.370 |
| DDIT3 | DNA-damage-inducible transcript 3 | 1.029 | 3.335 | 2.798 |
| CIT | citron rho-interacting serine/threonine kinase | 0.674 | 0.579 | 0.307 |
| GJB6 | gap junction protein beta 6 | 1.194 | 1.085 | 2.607 |
| GREM1 | gremlin 1, DAN family BMP antagonist | 2.648 | 1.654 | 3.789 |
| SMAD3 | SMAD family member 3 | 0.958 | 0.743 | 0.477 |
| PML | promyelocytic leukemia | 0.898 | 0.681 | 0.496 |
| GADD45B | growth arrest and DNA-damage-inducible, beta | 0.670 | 2.463 | 2.096 |
| RHOB | ras homolog family member B | 1.326 | 2.145 | 2.242 |
| BCL2L11 | BCL2-like 11 (apoptosis facilitator) | 0.938 | 0.722 | 0.493 |
| CYP1B1 | cytochrome P450, family 1, subfamily B, polypeptide 1 | 7.451 | 1.326 | 3.097 |
| TIA1 | TIA1 cytotoxic granule-associated RNA binding protein | 0.992 | 0.516 | 0.368 |
| PLAGL2 | pleiomorphic adenoma gene-like 2 | 1.160 | 0.935 | 0.496 |
| BLCAP | bladder cancer associated protein | 0.863 | 0.447 | 0.490 |
| HMOX1 | heme oxygenase 1 | 0.927 | 1.551 | 3.322 |
| H1F0 | H1 histone family, member 0 | 0.621 | 0.784 | 0.398 |
| EP300 | E1A binding protein p300 | 0.845 | 0.584 | 0.490 |
| PIM3 | Pim-3 proto-oncogene, serine/threonine kinase | 1.207 | 1.594 | 2.230 |
| TNFSF10 | tumor necrosis factor (ligand) superfamily, member 10 | 0.556 | 0.911 | 0.428 |
| ZNF346 | zinc finger protein 346 | 1.143 | 0.702 | 0.495 |
| NAIP | NLR family, apoptosis inhibitory protein | 0.812 | 0.361 | 0.427 |
| SERPINB9 | serpin peptidase inhibitor, clade B (ovalbumin), member 9 | 1.188 | 1.244 | 0.492 |
| PLAGL1 | pleiomorphic adenoma gene-like 1 | 0.585 | 0.402 | 0.401 |
| PEG10 | paternally expressed 10 | 0.713 | 0.698 | 0.471 |
| CASP2 | caspase 2 | 0.900 | 0.680 | 0.467 |
| TP53INP1 | tumor protein p53 inducible nuclear protein 1 | 0.815 | 0.694 | 0.337 |
| TGFBR1 | transforming growth factor, beta receptor 1 | 0.778 | 0.615 | 0.494 |
| NOTCH1 | notch 1 | 0.910 | 0.640 | 0.458 |
| KRT80 | keratin 80, type II | 0.487 | 0.886 | 0.493 |
| KRT34 | keratin 34, type I | 1.240 | 0.885 | 2.738 |
| KRT9 | keratin 9, type I | 1.223 | 1.504 | 2.131 |
| MKI67 | marker of proliferation Ki-67 | 0.541 | 0.691 | 0.349 |
| **Inflammation** | | | | |
| CSF1 | colony stimulating factor 1 (macrophage) | 0.823 | 0.724 | 0.438 |
| TNFRSF9 | tumor necrosis factor receptor superfamily, member 9 | 0.476 | 0.596 | 0.350 |
| HSPG2 | heparan sulfate proteoglycan 2 | 0.912 | 0.552 | 0.366 |
| PTGS2 | prostaglandin-endoperoxide synthase 2 (prostaglandin G/H synthase and cyclooxygenase) | 5.509 | 1.874 | 5.072 |
| CASP4 | caspase 4 | 1.408 | 1.496 | 2.053 |
| LRP1 | LDL receptor related protein 1 | 0.943 | 0.589 | 0.421 |
| NFE2L2 | nuclear factor, erythroid 2-like 2 | 0.711 | 0.429 | 0.491 |
| HMOX1 | heme oxygenase 1 | 0.927 | 1.551 | 3.322 |
| CXCL1 | chemokine (C-X-C motif) ligand 1 (melanoma growth stimulating activity, alpha) | 0.878 | 0.609 | 0.467 |
| CXCL10 | chemokine (C-X-C motif) ligand 10 | 0.717 | 0.863 | 0.497 |
| NDST1 | N-deacetylase/N-sulfotransferase (heparan glucosaminyl) 1 | 0.819 | 0.583 | 0.444 |
| NAIP | NLR family, apoptosis inhibitory protein | 0.812 | 0.361 | 0.427 |
| IKBKB | inhibitor of kappa light polypeptide gene enhancer in B-cells, kinase beta | 0.850 | 0.670 | 0.462 |
| NOTCH1 | notch 1 | 0.910 | 0.640 | 0.458 |
| MT1L | metallothionein 1L (gene/pseudogene) | 1.424 | 1.002 | 2.421 |
| MT1M | metallothionein 1M | 4.654 | 2.634 | 4.734 |
| MT1A | metallothionein 1A | 4.636 | 4.356 | 5.136 |
| MT1F | metallothionein 1F | 1.950 | 1.195 | 2.494 |
| MT1H | metallothionein 1H | 13.081 | 2.608 | 9.883 |
| **Immune response** | | | | |
| CSF1 | colony stimulating factor 1 (macrophage) | 0.823 | 0.724 | 0.438 |
| TLR4 | toll-like receptor 4 | 1.264 | 1.378 | 2.129 |
| QSOX1 | quiescin Q6 sulfhydryl oxidase 1 | 0.900 | 0.775 | 0.467 |
| TNFRSF9 | tumor necrosis factor receptor superfamily, member 9 | 0.476 | 0.596 | 0.350 |
| IFI6 | interferon, alpha-inducible protein 6 | 0.470 | 0.662 | 0.405 |
| NOTCH2 | notch 2 | 0.942 | 0.495 | 0.471 |
| RC3H1 | ring finger and CCCH-type domains 1 | 0.842 | 0.515 | 0.349 |
| TMEM63A | transmembrane protein 63A | 1.120 | 0.653 | 0.464 |
| IFIT3 | interferon-induced protein with tetratricopeptide repeats 3 | 0.380 | 0.793 | 0.489 |
| TRIM8 | tripartite motif containing 8 | 1.129 | 0.761 | 0.401 |
| CD44 | CD44 molecule (Indian blood group) | 1.122 | 0.286 | 0.285 |
| RBM14 | RNA binding motif protein 14 | 0.910 | 0.626 | 0.444 |
| IL18BP | interleukin 18 binding protein | 0.740 | 0.381 | 0.268 |
| CASP4 | caspase 4 | 1.408 | 1.496 | 2.053 |
| ITFG2 | integrin alpha FG-GAP repeat containing 2 | 0.715 | 0.459 | 0.497 |
| METTL7A | methyltransferase like 7A | 0.956 | 1.201 | 0.307 |
| LRP1 | LDL receptor related protein 1 | 0.943 | 0.589 | 0.421 |
| RAP1B | RAP1B, member of RAS oncogene family | 0.596 | 0.300 | 0.410 |
| APAF1 | apoptotic peptidase activating factor 1 | 0.714 | 0.599 | 0.370 |
| OAS3 | 2-5-oligoadenylate synthetase 3 | 0.867 | 0.616 | 0.422 |
| STAT2 | signal transducer and activator of transcription 2 | 0.691 | 0.625 | 0.451 |
| RGCC | regulator of cell cycle | 1.435 | 1.668 | 2.415 |
| TRAV38-1 | T cell receptor alpha variable 38-1 | 0.933 | 0.654 | 0.494 |
| IRF9 | interferon regulatory factor 9 | 0.487 | 0.688 | 0.478 |
| IGHJ1 | immunoglobulin heavy joining 1 | 1.329 | 1.690 | 2.113 |
| IGHV5-51 | immunoglobulin heavy variable 5-51 | 1.155 | 1.935 | 3.138 |
| IGHV1-58 | immunoglobulin heavy variable 1-58 | 1.664 | 2.690 | 2.714 |
| SMAD3 | SMAD family member 3 | 0.958 | 0.743 | 0.477 |
| PML | promyelocytic leukemia | 0.898 | 0.681 | 0.496 |
| IGF1R | insulin-like growth factor 1 receptor | 0.921 | 0.619 | 0.417 |
| SP2 | Sp2 transcription factor | 0.720 | 0.505 | 0.385 |
| STXBP4 | syntaxin binding protein 4 | 1.005 | 0.500 | 0.499 |
| SLC44A2 | solute carrier family 44 (choline transporter), member 2 | 0.840 | 0.830 | 0.446 |
| TARM1 | T cell-interacting, activating receptor on myeloid cells 1 | 0.998 | 1.336 | 2.110 |
| IGKV1OR2-108 | immunoglobulin kappa variable 1/OR2-108 (non-functional) | 2.046 | 0.267 | 0.426 |
| SIRPA | signal-regulatory protein alpha | 0.811 | 0.762 | 0.495 |
| SAMHD1 | SAM domain and HD domain 1 | 0.883 | 0.754 | 0.425 |
| SLPI | secretory leukocyte peptidase inhibitor | 1.149 | 1.568 | 2.133 |
| IGLV1-50 | immunoglobulin lambda variable 1-50 (non-functional) | 0.999 | 1.433 | 2.032 |
| IGLV3-10 | immunoglobulin lambda variable 3-10 | 1.951 | 2.027 | 3.364 |
| OSM | oncostatin M | 0.928 | 0.821 | 2.096 |
| PARP14 | poly(ADP-ribose) polymerase family member 14 | 0.704 | 0.581 | 0.388 |
| PLD1 | phospholipase D1, phosphatidylcholine-specific | 0.806 | 0.634 | 0.483 |
| TNFSF10 | tumor necrosis factor (ligand) superfamily, member 10 | 0.556 | 0.911 | 0.428 |
| TNK2 | tyrosine kinase, non-receptor, 2 | 0.834 | 0.542 | 0.380 |
| CXCL1 | chemokine (C-X-C motif) ligand 1 (melanoma growth stimulating activity, alpha) | 0.878 | 0.609 | 0.467 |
| CXCL10 | chemokine (C-X-C motif) ligand 10 | 0.717 | 0.863 | 0.497 |
| DDX60 | DEAD (Asp-Glu-Ala-Asp) box polypeptide 60 | 0.652 | 0.547 | 0.463 |
| NAIP | NLR family, apoptosis inhibitory protein | 0.812 | 0.361 | 0.427 |
| SLCO4C1 | solute carrier organic anion transporter family, member 4C1 | 1.197 | 0.775 | 0.343 |
| BTN3A2 | butyrophilin, subfamily 3, member A2 | 0.761 | 0.730 | 2.392 |
| FBXO9 | F-box protein 9 | 0.944 | 0.813 | 0.479 |
| FBXO9 | F-box protein 9 | 1.085 | 0.330 | 0.324 |
| ULBP2 | UL16 binding protein 2 | 1.348 | 2.174 | 2.218 |
| IGF2R | insulin-like growth factor 2 receptor | 1.007 | 0.620 | 0.452 |
| SERPINB9 | serpin peptidase inhibitor, clade B (ovalbumin), member 9 | 1.188 | 1.244 | 0.492 |
| HIST1H2BJ | histone cluster 1, H2bj | 0.671 | 0.537 | 0.324 |
| TRIM26 | tripartite motif containing 26 | 0.836 | 0.804 | 0.494 |
| TAPBP | TAP binding protein (tapasin) | 0.917 | 0.802 | 0.431 |
| TRIM56 | tripartite motif containing 56 | 1.096 | 0.735 | 0.489 |
| TRBV6-5 | T cell receptor beta variable 6-5 | 0.715 | 1.682 | 2.050 |
| ZC3HAV1 | zinc finger CCCH-type, antiviral 1 | 0.693 | 0.582 | 0.341 |
| IKBKB | inhibitor of kappa light polypeptide gene enhancer in B-cells, kinase beta | 0.850 | 0.670 | 0.462 |
| HGSNAT | heparan-alpha-glucosaminide N-acetyltransferase | 0.769 | 0.626 | 0.422 |
| PDCD1LG2 | programmed cell death 1 ligand 2 | 1.320 | 1.063 | 2.307 |
| PRSS3 | protease, serine, 3 | 1.256 | 1.303 | 2.065 |
| NOTCH1 | notch 1 | 0.910 | 0.640 | 0.458 |
| HUWE1 | HECT, UBA and WWE domain containing 1, E3 ubiquitin protein ligase | 0.972 | 0.618 | 0.477 |
| RPL39 | ribosomal protein L39 | 1.080 | 1.882 | 2.009 |
| PSMD3 | proteasome 26S subunit, non-ATPase 3 | 1.181 | 0.392 | 0.394 |
| *SOD1* | superoxide dismutase 1, soluble | 0.956 | 1.503 | 1.577 |
