# Supplementary material for: Urban Particles Elevated Streptococcus pneumoniae Biofilms, Colonization of the Human Middle Ear Epithelial Cells, Mouse Nasopharynx and Transit to the Middle Ear and Lungs
Source: Sci Rep. 2020 Apr 6;10:5969. doi: 10.1038/s41598-020-62846-7 (PMC7136263; doi:10.1038/s41598-020-62846-7)
Supplement: Supplementary file 7 — Supplementary table III. [file 41598_2020_62846_MOESM7_ESM.docx]

SUPPLEMENTRY TABLE III. Conformation of Microarray Gene Expression Fold Change in RT PCR

| **Gene name** | **Protein encode** | **Real-time PCR** | | |
| --- | --- | --- | --- | --- |
|  |  | UP | *S. pneumoniae* | Co-treatment |
| HMOX1 | heme oxygenase 1 | 1.2 | 1.7 | 2.9 |
| PTGS2 | prostaglandin-endoperoxide synthase 2 (prostaglandin G/H synthase and cyclooxygenase) | 3.2 | 1.9 | 5.7 |
| IL24 | interleukin 24 | 2.6 | 3.80 | 4.15 |
| *SOD1* | superoxide dismutase 1, soluble | 1.3 | 1.5 | 2.1 |
| CYP1B1 | cytochrome P450, family 1, subfamily B, polypeptide 1 | 4.9 | 5.8 | 4.10 |
| MKI67 | marker of proliferation Ki-67 | 0.51 | 0.54 | 0.27 |
| CSF1 | colony stimulating factor 1 (macrophage) | 0.52 | 0.65 | 0.40 |
| CD44 | CD44 molecule (Indian blood group) | 0.95 | 0.34 | 0.31 |
| IFI6 | interferon, alpha-inducible protein 6 | 0.31 | 0.48 | 0.39 |
| BCL2L11 | BCL2-like 11 (apoptosis facilitator) | 0.58 | 0.49 | 0.36 |
